# Prevalence of psychological distress among parents of children with intellectual disabilities in Malawi

**DOI:** 10.1186/s12888-018-1731-x

**Published:** 2018-05-24

**Authors:** Charles Masulani-Mwale, Felix Kauye, Melissa Gladstone, Don Mathanga

**Affiliations:** 1 St. John of God Mental Health Services, Malawi, Box 744, Mzuzu, Malawi; 2 Department of Community Health, University of Malawi College of Medicine, P/Bag 860, Blantyre, Malawi; 3 UK Department of Women and Children’s Health, Institute of Translational Medicine, University of Liverpool, Alder Hey Children’s NHS Foundation Trust, Liverpool, UK

**Keywords:** Prevalence, Psychological distress, Parents, Intellectual disabilities

## Abstract

**Background:**

Children with intellectual disabilities are common and are increasing in number as more children survive globally. In stark contrast to the 1–3% prevalence of intellectual disability in children globally (reported by WHO), studies from Malawi provide alarmingly high rates (26%). We know that the prevalence of psychological distress is as high as 50% in parents of children with intellectual disabilities in Europe and the US. No such studies have yet been conducted in Africa. This study is aimed at determining the prevalence and risk factors for psychological distress among parents of intellectually disabled children in Malawi.

**Methods:**

This quantitative cross-sectional study was conducted in January and February 2015. One hundred and seventy mothers and fathers of children with intellectual disabilities as diagnosed by psychiatric clinical officers were randomly sampled from two selected child disability clinics. The Self-Reporting Questionnaire (SRQ) was used “as measure for psychological distress and questions on socio-demographic variables were administered to all consenting participants.” Data was coded, cleaned and analyzed using STATA.

**Results:**

70/170 (41.2%) of parents of children with intellectual disabilities reported psychological distress. Univariate and multivariate analysis showed that area of residence (*P* < 0.05), low socio-economic status (*P* < 0.05), knowledge of the disability of one’s child (*P* < 0.05), low confidence in managing the disabled child (*P* < 0.05), increased perceived burden of care (*P* = 0.05), and having no sources for psychological support (*P* < 0.05) significantly predicted psychological distress among the parents for children with disabilities.

**Conclusion:**

There is huge burden of psychological distress among parents of intellectually disabled children in Malawi. Psychosocial interventions are urgently needed to support parents of children with intellectual disability in Malawi.

## Background

Intellectual disabilities, characterized by limitations in intellectual functioning as well as adaptive behaviors, are very common in children worldwide, with rates likely to increase as more children survive due to improved medical care [1, 2]. The prevalence of intellectual disability among children in developing countries is estimated as being particularly high [3]. Rates of neurological disability in children Kenya, which includes intellectual disability, has been estimated to be as much as 9.3% [4] whilst in Malawi, a recent key informant study estimated 31% percent of children having a disability, with 26% of them having an intellectual disability [5].

The relationship between caring for these children and psychological distress for their parents has been studied but mainly in developed world settings [6–9]. Some studies have been conducted in Low and Middle Income Countries (LMIC] such as Kenya, Kuwait, Qatar and India which similarly report rates of 47–50% prevalence of psychological disorders amongst these parents [2, 10–12]. Parenting such children may lead to difficulties with family functioning, parenting stress, and different parenting style compared to parenting normally developing children [13]. The effect of stress on family functioning can also be related to negative couple attribution related to marital satisfaction [14]. It can also lead to divorce and financial problems [15–17]. Many experts believe that much of this psychological distress is related to the stigma which surrounds caring for a disabled child as well as lack of knowledge of its causes.

Several studies highlight the child, parental and environmental factors which predict psychological distress amongst parents of intellectually disabled children. Factors include: lower socioeconomic status [18], single motherhood [11, 19], feminine gender [20], perceived burden of care [21], lack of psychosocial support [19], knowledge of child’s disability [22] and having more than one child with a disability in the family [12]. Furthermore, having a younger child and some specific disorders such as autism are more likely to cause high psychiatric symptomatology among parents [18]. While most of these studies have been conducted in the developed world, few studies have been done in Africa where disability estimates are high and services are almost non-existent. It is important that the extent of this problem is explored so that necessary strategies and interventions can be instituted in these settings in order to support parents better in these settings. This paper describes the prevalence and factors associated with psychological distress among parents of intellectually disabled children attending disability clinics in Malawi, a low income country.

## Methods

### Design and setting of the study

This cross-sectional study was conducted between January and February 2015. Study participants were mothers and fathers of children with intellectual disabilities attending services at St John of God disability clinics in Mzuzu and Children of Blessing disability center in Lilongwe in Malawi. St John of God conducts disability clinics in all urban and semi-urban townships in Mzuzu whilst the Children of Blessing disability center offers its services in a semi-urban township in area 25 in Lilongwe city. Parents of children who screened positive for intellectual disability (meeting DSM-IV-TR criteria for intellectual disability, as measured by a psychiatric clinical officer, were enrolled in this study.

Parents were included in this study if they were 1) aged 18 years and above; 2) were the main carers for an intellectually disabled child; 3) their child had a diagnosis of intellectually disability according to DSM V-TR criteria (Intellectual and adaptive functioning deficits in conceptual, social, and practical domains) as diagnosed by a psychiatric clinical officer and 4) gave consent to participate in the study.

### Data collection

Five data collectors (including the lead author and four qualified nurses) collected quantitative data at the two clinics. The nurses were given 3 days training in study methodology and measurement of psychological distress as well as its risk factors. We verbally administered the study questionnaire to consenting participants in rooms that provided privacy over a period of 15 to 20 min. We collected demographic details of the participants which from previous studies have been significantly associated with psychosocial distress. This included urban vs. rural site, age which in a subsequent was compressed into two categories of vulnerability (Vulnerable = too young/too old to parent [because they would struggle to care for the child due to young age or old age] versus less vulnerable = middle aged parents [who are productive and have the energy to provide care]), sex (male versus female), socio-economic status (SES) through wealth rankings, occupation, education status and religious affiliation of parent. We also collected data from the Self-Reporting Questionnaire (SRQ) as the measure of psychological distress. Finally, we asked a series of other questions which are associated with better or worsening psychological distress in parents of children with disabilities. This included; a) availability of psychological support [availability of counselling or accompaniment received from professionals, religious and community leaders who are supportive of participants’ psychological and mental health concerns, and respond appropriately as needed [23] based on Protégé–mentor agreement psychosocial support by Noe’s 14 item-Psychosocial Support mentoring scale of 1988], b) knowledge of child’s disability and c) perceived confidence in managing the disabled child and perceived burden of care.

The SRQ is a brief measure of psychiatric symptoms designed by the WHO to be used across cultures [24]. It has been well validated translated and back translated for use in Malawi [25]. This includes a recent study validating it against Structured Clinical Interview for DSM-IV (SCID) for major depressive episodes in mothers of infants attending for measles vaccination in Thyolo District Hospital-Malawi [25]. In this study, SRQ had high internal consistency (Cronbach’s alpha 0.85], and the area under the ROC curve for detection of current major depressive disorder was 0.856 [95% CI 0.813 to 0.900], while for current major or minor depressive disorder was 0.826 {95% CI 0.783 to 0.869} [25]. Our study has therefore adopted the 7/8 cut-off-point because of good epidemiological indices for major/minor depression [Sensitivity = 59.2, specificity = 85.4, positive predictive value = 64.1 and negative predictive value = 82.7]. The SRQ is scored out of 20. Participants who scored 8 and above were considered distressed while those scoring 7 and below were considered non-distressed.

“We computed Cronbach’s alpha using SPSS command and this was 0.794. This suggests that the internal consistency is between adequate and good”.

### Statistical analysis

The collected data was coded on a computer, cleaned and analyzed using STATA version 13.0 software (Stata Corp., College Station, TX, USA). Cases were defined as those scoring 7 or above on the SRQ. The total number of cases was divided by total number of subjects to give the prevalence. The level of significance was set at *P* < 0.05. The association between psychological distress and the socio-demographic variables (in section “Background” of the questionnaire) and other factors associated with psychological distress (availability of psychological support, knowledge of child’s disability and perceived confidence in managing the disabled child) were computed using a chi-square test. All variables that had a significant association with psychological distress in the initial univariate analysis were placed into the multivariate analysis. A multiple logistic regression was then conducted to check significance of confounding variables which were found significant in the univariate analysis.

## Results

Of the 186 participants who were sampled for the study, 184 (representing 98.9% of the participants) participated in the study. Of the total number of 186 participants, 170 (91%) had complete data sets.

### Socio-demographic features

47.3% (88) of parents were from Lilongwe (an urban setting) while the rest were from Mzuzu (a somewhat more rural setting). The mean age of the participants was 34.4 years (SD: 9.5). In a subsequent analysis, where age was compressed into two categories of vulnerability, it was found that 21% [26] of the participants were young/too old to parent (vulnerable) while 79% (147) were middle aged. The majority of participants were female (90.1% (*n* = 156)) with 79% (*n* = 147) of the participants married. The majority of participants (103 (55.7%)) had completed secondary education whilst 64 (34.6%) had also completed their tertiary education. A 62.4% of the participants were unemployed (*n* = 116) and a 38% (*n* = 70) of the participants had a high socioeconomic status whilst 17.4% middle and 44.6% low socioeconomic status respectively. In the subsequent analysis this variable was compressed into two socio-economic profiles for easy analysis (low versus upper). The majority of participants (127 (68.3%)) indicated that they had no psychological support; whilst 59 (31.7%) had the required support.

### Prevalence of psychological distress on the SRQ

The prevalence of psychological distress among participants was 41.2% (70/170). The factors which were associated psychological distress among the participants were; attendance at Lilongwe center (χ^2^ = 11.7; df = 1; *P* < 0.001); low socio-economic status (χ^2^ = 4.9; df = 1; *P* < 0.025); knowledge of the disability of one’s child (χ^2^ = 4.8; df = 1; P < 0.02); low confidence in managing the disabled child (χ^2^ = 10.4; df = 1; *P* < 0.001); increased perceived burden of care (χ^2^ = 8.2; df = 1; *P* < 0.004); and having no sources for psychological support (χ^2^ = 4.03; df = 1; *P* < 0.045 (see Table 1). Other variables such as participants’ age, sex, education, marital state, spiritual support, relationship with the child, and occupation did not have a significant association with psychological distress among the participants.

**Table 1:** Results of univariate and multivariate analyses on variables tested for associations with psychological distress

Variable		Crude risk ratios		Adjusted risk ratios	
	Category	Crude RR (CI)	*P* value	Adjusted RR (CI)	*P* value
Center	Lilongwe	1.0		1.0	
	Mzuzu	0.33 (0.17–0.6)	0.001*	0.45 (0.22–0.93)	0.03*
Socio-economic status	Low SES	1.0		1.0	
	Upper SES	0.49 (0.2–0.9)	0.02*	0.69 (033–1.4)	0.32
Sex	Female	1.0		1.0	
	Male	1.16 (0.43–3.1)	0.76	1.17 (0.37–3.6)	0.77
Knowledge of one’s child’s disability	No	1.0		1.0	
	Yes	2.5 (1.08–6.13)	0.03*	2.2 (0.87–5.7)	0.09
Confidence in managing own child	No confidence	1.0		1.0	
	Has confidence	0.35 (0.18–0.66)	0.001*	0.42 (0.2–0.8)	0.02*
Perceived burden of care	Low	1.0		1.0	
	High	2.7 (1.3–5.6)	0.005*	1.7 (0.8–3.8)	0.151
Psychological Support	No	1.0		1.0	
	Yes	0.5 (0.25–0.98)	0.04*	0.56 (0.25–1.2)	0.15

1.0 = Reference

*p < 0.05

All variables found significant (*p* < 0.05) in the univariate analyses (location, SES, knowledge of the disability of one’s child, low confidence in managing the disabled child, increased perceived burden of care and having no sources for psychological support) plus common confounders (age and sex)) were included in a multivariate logistic regression analysis. After adjusting for these variables; attendance at Lilongwe center (*p* < 0.05), and low confidence in managing the disabled child (*p* < 0.05), were still significantly correlated with psychological distress. Low socio-economic status, knowledge of the disability of one’s child, increased perceived burden of care, and having no sources for psychological support were not significant (Table 1).

## Discussion

The prevalence of psychological distress in our present studies was high with 41% reporting a level of psychological distress above the expected norm on the SRQ. This data clearly demonstrates that there are many undiagnosed and unmanaged psychological issues among parents of children with intellectual disabilities. Previous studies have reported prevalence of psychological distress ranging from 32 to 89% among parents of children with disabilities [10, 27]. The prevalence rate found in our study is high and reflects the magnitude of undiagnosed psychological distress not previously identified. This is compounded by the lack of psychological support provided by health facilities for these families in Malawi particularly with few trained health care providers in mental health in this setting. This finding highlights the need to better address the psychological issues among parents of children with intellectual disabilities by health care providers. The high prevalence observed in this study is consistent with previous studies. Rates found in our study are lower than those from Kenya where a rate of 79% prevalence is reported [10, 28]. This may be due differences in data collection instruments. Our study used SRQ which depends on participants’ self-report and may lead to under reporting bias.

On social demographic factors for the study participants, many of them were around thirties. This is mainly due to reproductive and caring age which is among the middle aged as found in similar studies [29, 30]. Many more women than men took part in this study. In Malawi, women are still the main carers for children and single motherhood is more common in mothers of children with disabilities as a result of marital breakdown caused by the birth of the disabled child in the family [31]. In some cases, husbands disassociate from the child with disability and the mother is blamed for bearing the disabled child [32]. The burden of care of all children with disabilities in Malawi tends to rest with females [32], even if other carers from the extended family such as grandmothers, aunts and uncles and older siblings might help. It is clearly important to introduce programs that particularly benefit women in enabling them to more confidently care for their child. It is also important to design and facilitate group-based psychological interventions which address issues affecting mainly women.

In this present study, the majority of participants sampled were unemployed and about 44% had a low socio-economic status. The relationship between development of a child’s disability and poverty is clear and likely to be the case in our group of participants [33]. This relationship can be explained by a number of factors related to unemployment and poverty. Firstly, the nutritional status of mothers during pregnancy can affect brain development but also make mothers more susceptible to infections and insults during pregnancy which all can affect the baby’s brain development. Secondly, many poor mothers are less likely to access health services due to unaffordable transportation and medication costs. This can compromise the health of the mothers and lead to delays in accessing perinatal services resulting in poor birth outcomes such as low birth weight and child asphyxia, both major predictors of child intellectual disability. A study on children with disabilities in Uganda found that families with a disabled child are more likely to live in poverty with up to 88% of caregivers finding it difficult to meet the basic needs of their children with disabilities and their families [34].

In our study, those families living in Mzuzu; belonging to upper socio-economic class, those with better parent education, and those having psychological support were associated with less psychological distress. Sex of the parent was not useful. Much of these factors may be related to the economic circumstances of the study population. Mzuzu population is more educated despite being a bit rural and hence people would have better livelihoods because they would be in better employment compared to Lilongwe. Studies have demonstrated that individuals with good socio-economic status and better parent education are more likely to seek and receive better support both materially and psychologically [35, 36]. This may be due to an increased capacity to travel and visit centers, to understand child disability issues and seek the required supports and hence have less unmanaged psychological burden resulting in low psychological distress among participants belonging to these classes.

Perceived high burden of care for the child was associated with high psychological stress in this study probably due to single-handed parenthood. This could also be to the declining of informal sources of support such as friends and religious groups that have been associated with reduced stress in carers of children with disabilities [26, 37–39]. Having low confidence in managing the disabled child was associated with more psychological distress. This is in line with similar studies that have shown that low confidence reduces parental self-efficacy in managing the child ending up stressing the parents and entire family [40, 41].

One of the most concerning factors in our study was that knowledge of one’s child disability was associated with increased psychological distress. This may be because parents who know about their child’s disability, focus more thoughts and attention on their child’s disability and its impact rather than focusing on the positive aspects of the child [42, 43]. Upon realizing that the child has an intellectual disability, which is incurable, thoughts and preoccupations about the disability may build up, and end up exacerbating distress among the parents [44]. This is important to consider when creating programs to support parents.

Finally, while feminine gender is a known predictor for distress [45], it was not significantly associated with psychological distress in this study. This could be attributed to the negligible sample size of men who participated in this study.

Our study has several strengths as well as a few issues to be considered. Our team has used a validated data collection instrument, one of only a few well validated for use in a sub-Saharan setting. Our study had an excellent response rate of 98.9% with almost all participants willing to take part in the study. Our study is the first to be conducted Malawi and the first one to have a definitive diagnosis of intellectual disability (through the use of the DSM-IV-TR) among all children of study participants.

The main limitation of our study was that it was center-based and gives only a reflection of prevalence rates of psychological distress in caregivers of children who are coming to a center. It is very possible that there are higher numbers of parents with psychological distress who may not bring their children to clinics and that the rates are actually higher. On the opposite front, it may be that parents of children with disabilities who suffer from psychological distress are more likely to come to a center, making the rates in our study higher than they would be in the community. We would not be able to gain this information without doing a community-based study. Secondly, is very difficult to determine the causal inference on whether caring the disabled child preceded the psychological distress as the data was collected at one point in time. Finally, as mentioned above, the use of parent self-report too for assessing psychological distress may not be a most reliable measure psychological distress due to over or under reporting by the participants [46, 47]. An observational study would be ideal but difficult to achieve. Finally, variables such as the type of intellectual disability (definitive clinician diagnosis) and the severity of the condition were not considered in the study and may have a significant impact on the parent’s psychological distress.

The generalization of these findings may also be limited to urban settings and surrounding environs because the study did not sample participants from the very rural and poor population where the prevalence may be higher due to the higher rates of socio-economic challenges.

### Implication for practice and future research

Our study has demonstrated the high burden of psychological distress among parents of intellectually disabled children. This clearly highlights the need for the creation of interventions to support parents of children with disabilities which target the mental health and psychosocial well-being of parents. Our research has also highlighted the need for parents of children with disabilities in Malawi to be provided with education on childhood disability. Future studies which provided further evidence on the risk factors related to psychological distress for parents may be helpful. This would include studies which measured the functional severity of the child’s disability as well as the specific diagnostic conditions which are more linked with needing parental input and support.

## Conclusion

There is huge burden of psychological distress among parents of intellectually disabled children in Malawi. The factors associated with this distress include: residence in Lilongwe, low socio-economic status, knowledge of the disability of one’s child, low confidence in managing the disabled child, increased perceived burden of care, and having no sources for psychological support. Contextualized psychosocial interventions need to be developed for supporting parents of children with intellectual disability in Malawi.

## Abbreviations

CI: Confidence interval
DSM-IV-TR: Diagnostic and Statistical Manual of Mental Disorders-Fourth Edition (Text Revision)
LAMIC: Low and Middle Income Country
RR: Risk ratio
SES: Socioeconomic status
SRQ: Self-Reporting Questionnaire
